# Polymer Injectivity Test Design Using Numerical Simulation

**DOI:** 10.3390/polym12040801

**Published:** 2020-04-03

**Authors:** Mohamed Adel Alzaabi, Jørgen Gausdal Jacobsen, Shehadeh Masalmeh, Ali Al Sumaiti, Øystein Pettersen, Arne Skauge

**Affiliations:** 1 Department of Chemistry, University of Bergen, 5007 Bergen, Norway; joja@norceresearch.no (J.G.J.);; 2 Norwegian Research Center, 5008 Bergen, Norway; oype@norceresearch.no; 3 Abu Dhabi National Oil Company, P.O. Box 898 Abu Dhabi, UAE; smasalmeh@adnoc.ae (S.M.); aalsumaiti@adnoc.ae (A.A.S.); 4 Energy Research Norway, 5007 Bergen, Norway

**Keywords:** chemical EOR, polymer flooding, in situ rheology, polymer injectivity, polymer modeling

## Abstract

Polymer flooding is an enhanced oil recovery (EOR) process, which has received increasing interest in the industry. In this process, water-soluble polymers are used to increase injected water viscosity in order to improve mobility ratio and hence improve reservoir sweep. Polymer solutions are non-Newtonian fluids, i.e., their viscosities are shear dependent. Polymers may exhibit an increase in viscosity at high shear rates in porous media, which can cause injectivity loss. In contrast, at low shear rates they may observe viscosity loss and hence enhance the injectivity. Therefore, due to the complex non-Newtonian rheology of polymers, it is necessary to optimize the design of polymer injectivity tests in order to improve our understanding of the rheology behavior and enhance the design of polymer flood projects. This study has been addressing what information that can be gained from polymer injectivity tests, and how to design the test for maximizing information. The main source of information in the field is from the injection bottom-hole pressure (BHP). Simulation studies have analyzed the response of different non-Newtonian rheology on BHP with variations of rate and time. The results have shown that BHP from injectivity tests can be used to detect in-situ polymer rheology.

## 1. Introduction

Polymer flooding is a well-established chemical enhanced oil recovery (CEOR) method that has been widely used for more than half a century. It was initially introduced to alleviate the issues related to unfavorable mobility ratio, induced by reservoir heterogeneity and/or high oil viscosity. These issues were remediated by adding polymers to the injected water to improve overall aerial and volumetric sweep efficiency [1]. The main mechanisms of polymer flooding are sweep improvement that consists of mitigating viscous fingering and improving crossflow between vertical heterogeneous layers [2,3]. In addition, numerous studies indicate that non-Newtonian polymer solutions can improve microscopic displacement efficiency and may reduce water-flood residual oil saturation [3,4].

The most commonly used polymer for CEOR applications is synthetic polymer partially hydrolyzed polyacrylamide (HPAM), with a typical hydrolysis degree range of 15%–33%. In bulk flow, viscosity measurements from rheometers show that HPAM exhibit shear thinning behavior, which can be explained by the disentanglement and realignment of polymer coils as velocity increases in the laminar flow regime. In addition, numerous polymer flow experiments in porous media have shown that HPAM exhibits an apparent shear thickening behavior beyond a critical shear rate [5]. Consequently, apparent viscosity attains a maximum value in the immediate near-wellbore region. This viscosity increment is often referred to as extensional or elongational viscosity as it is attributed to the extensional flow regime of the polymer. Both the coil-stretch theory and transient network theory has been suggested to account for the extensional flow phenomenon. According to the coil-stretch theory, which is adhered to by the authors of this paper, the flexible coiled molecules of HPAM experience stretching, entanglement and collisions at high shear, which results in a larger flow resistance, i.e., apparent shear thickening behavior. This behavior is a special property of elastic polymers in flow through porous media and is not observed for biopolymers such as xanthan [6].

Injectivity is one of the most important parameters in the design of any reservoir flooding application. Failure in estimating injectivity within acceptable error margins can have a significant impact on the expected recovery increment and thus on the economic feasibility of the project. For polymer flooding applications, accurate estimation of injectivity is more significant due to the polymer high viscosity and its non-Newtonian behavior. This behavior may result in the well operating near (or under) formation fracturing conditions, which can significantly affect in-situ polymer rheology.

Several important observations have been made in recent years that may explain the deviation between field injectivity results compared to initial expectations. Skauge et al. demonstrated that onset of shear thickening occurs at significantly higher velocities in radial compared to linear flow [7]. In addition, the extent of shear thinning was more pronounced in radial flow, while the extent of shear thickening was reduced compared to linear flow. The presence of residual oil is another factor that may have a significant impact on polymer in-situ rheology. Authors of [5] observed a significant reduction of polymer in-situ viscosity in the presence versus absence of residual oil. Furthermore, it was demonstrated through experimental work that the preshearing polymer before injection helps in improving injectivity by reducing elastic properties of the polymer while maintaining viscous properties, i.e., reducing or eliminating the extensional shear thickening behavior at high shear rates near the well-bore [8].

### 1.1. Modeling and Simulation of Polymer Injectivity

Numerical simulation is an essential tool for the assessment of polymer flooding lab results and fundamental theory. It is also important for designing polymer field projects as well as predicting the performance and outcomes of the project.

One of the early attempts to model polymer flooding was by Zeito (1968) [9]. He created a 3D numerical simulation to predict the performance of polymer flooding in any type of reservoir. His model, however, was missing the component of in-situ non-Newtonian behavior of polymer, which was later found to be fundamental in polymer flooding mechanisms. Bondor et al. (1972) added the polymer in-situ rheology impact through using modified Blake-Kozeny power law for fluids [10]. They also included the impact of other factors such as permeability reduction and non-linear mixing of polymer and water. Seright (1983) developed an analytical model for injectivity in radial coordinates [11]. His model combined a mechanical degradation correlation, linear core flood results of resistance factor and Darcy equation in radial flow, to calculate total injection pressure drop. Recently, Lotfollahi et al. (2016) have proposed an injectivity model to simulate polymer injectivity decline in both laboratory and field tests [12]. Their model coupled the effects of deep-bed filtration and external filter-cake formation caused by polymer adsorption/retention, to the viscoelastic polymer rheology. They also emphasized the advantage of using radial coordinates with a fine gridding scheme to reduce the error of velocity calculation in the near-wellbore area and hence capture polymer rheology more accurately. Some commercial reservoir simulation software have also included modules to model polymer flooding such as STARS of Computer Modeling Group Ltd. (CMG), ECLIPSE 100 of Schlumberger, and REVEAL of Petroleum Experts [13]. The simulator used in this study is STARS of CMG, which includes polymer modules that accounts for polymer rheology dependence on the shear rate or velocity, polymer adsorption, permeability reduction and impact of polymer concentration and salinity on viscosity.

### 1.2. Paper Objective

This study aims to utilize numerical simulation of polymer flooding on both lab and field scales in order to optimize the design of field polymer injectivity tests. The main objective is to analyze the relationship between the injector bottom-hole pressure (BHP) and polymer in-situ rheology. Beside rheology, the impact of simple heterogeneity is also investigated along with the impact of permeability reduction because of polymer adsorption in low-permeability layers. This is to simplify the process of interpreting field tests data, since the sole source of data in the field is usually BHP variations with time and BHP as a function of different injection rates.

## 2. Materials and Methods (Lab Scale)

**Figure 1:**
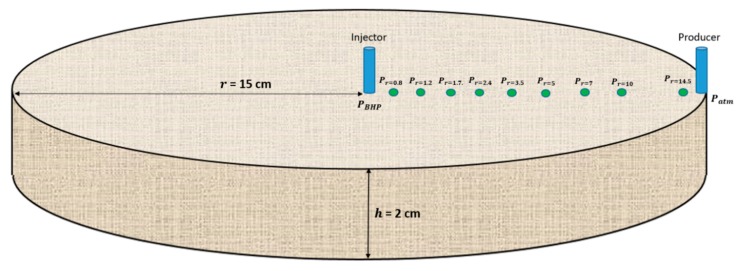
Illustration of the circular Bentheimer disc with pressure ports.

The radial flow experiment history matched in this paper was performed on a circular Bentheimer disc (radius = 15 cm, thickness = 2 cm, injection well radius = 0.325 cm and porosity = 0.25). Before oil was introduced, absolute permeability was measured and was equal to 2200 mD. The sample was prepared according to the method described in the literature for circular Bentheimer discs with internal pressure taps [6,7]; including ageing with heavy crude oil, followed by brine flooding to residual oil saturation of 0.34. Pressure ports were mounted both internally and in an injection well and producer, as depicted in Figure 1.

A relatively low salinity brine (7000 ppm TDS) was used in this study, with a viscosity of 1.02 cP at 22 °C. A 1000 ppm HPAM polymer solution was used (Flopaam 3330S, 8 MDa, 30% hydrolysis, SNF Floerger) and prepared according to the API method (RP 63, 1990, American Petroleum Institute). Shear viscosity of the polymer solution was measured to be 11.5 cP at 10 s^−1^, with zero shear viscosity extrapolated to 13.9 cP. It was concluded that negligible mechanical degradation occurred based on injected versus effluent viscosity measurements.

Initially, the brine solution was injected at ten different flow rates (0.05–2 mL/min) and effective permeability to brine ($k_{b,init}$) was calculated from Darcy’s law for radial flow:

$$k_{b,init}=-\frac{\mu Q}{2\pi h\Delta P}\ln\frac{r_{i}}{r}$$

where μ is brine viscosity, Q is volumetric injection rate, h is disc thickness, ΔP is the pressure drop between a specified port at radius $r_{i}$ and the producer at r.

Following the initial brine flood, a 1000-ppm HPAM solution was injected at similar injection rates (0.05–2.0 mL/min). Before measurements began, the polymer solution was injected at 0.1 mL/min for at least two pore volumes to ensure that retention was satisfied. Tapering was also performed and the final step of the radial polymer flood experiment consisted of a final brine flood to determine the permeability after the polymer flood and to calculate the residual resistance factor (RRF), which was equal to 1.2 for this experiment. Since permeability values obtained from initial brine flood was used in simulations, apparent viscosity is equal to resistance factor (RF) in this paper, where RF is defined as:

$$RF=\frac{\Delta P_{p}}{\Delta P_{b,init}}$$

where $\Delta P_{p}$ is the pressure drop during polymer flow and $\Delta P_{b,init}$ is the pressure drop during brine flow before polymer was introduced to the porous media.

### Simulation of Radial Flow Experiments

A radial grid with 360 sectors constituted the simulation model. Each of these sectors consist of 150 grid blocks, where the grid block cell size was 1 mm. Sensitivity analysis showed a negligible accuracy improvement when reducing the grid block size below 1 mm. Residual oil saturation after the brine flood resulted in a non-uniform oil saturation profile between the injector and producer. The history match of the brine differential pressure between internal pressure ports and the producer enabled determination of local permeabilities. Since Bentheimer sandstone is assumed to be homogeneous, the average effective permeability was used together with local permeabilities to calculate correction factors accounting for the non-uniform oil saturation.

## 3. Results and Discussion (Lab Scale)

**Figure 2:**
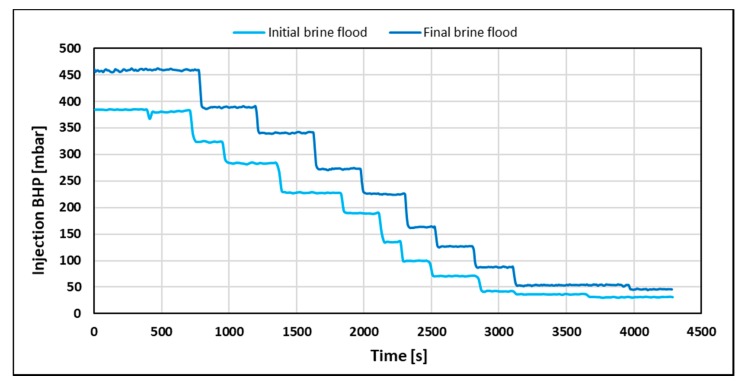
Injection bottom-hole pressure (BHP) versus time for initial (prior to polymer flood) and final brine flood (after polymer tapering).

Average effective permeability was 33.8 and 28 mD using the differential pressure response from the initial and final brine flood, respectively. Injection BHP build-up was also recorded for each individual injection rate during both brine floods as shown in Figure 2.

**Figure 3:**
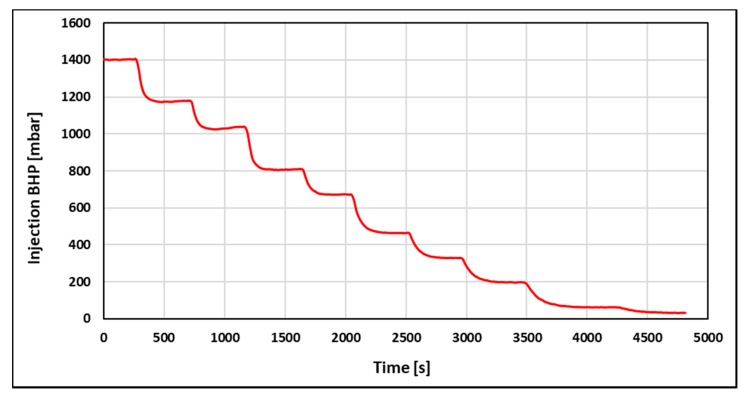
Pressure build up for the 1000-ppm polymer flood.

Injection BHP stabilization time was independent of volumetric injection rate and equal to 40 s for both brine floods. However, pressure stabilization was expected to occur instantaneously when injecting a Newtonian fluid. Thus, it was concluded that the system had a delay of 40 s. The delay was attributed to incomplete pressure communication due to low values of counter pressure from the production line (2–4 mbar). To investigate if stabilization time is dependent on polymer rheology, the injection BHP build-up for the polymer (Figure 3) was also recorded.

Injection BHP build-up from the polymer flood was clearly distinguishable from the Newtonian pressure response obtained from brine floods. Firstly, pressure stabilization time during the polymer flood was significantly higher (3–9 times) than for brine floods. In addition, stabilization time increased monotonically with decreasing volumetric injection rate (from 140 at 2 mL/min to 360 s at 0.05 mL/min). This suggests that the polymer rheology behavior is different at low compared to high injection rates.

To quantitatively analyze the polymer rheology of the 1000-ppm HPAM solution in the presence of residual oil, stabilized polymer pressure response was history matched as a function of both the volumetric injection rate, dP(Q), and radial distance, dP(r):

- dP(Q): Analogue to conventional polymer rheology estimation from field data, pressure drop across the entire disc (injection BHP) is history matched as a function of the volumetric injection rate, yielding a single rheology curve. Since injection BHP is influenced by near-well effects such as skin and mechanical degradation, the robustness and accuracy of this method may be debatable.
- dP(r): Using only internal pressure ports, the pressure drop between individual ports and the producer is history matched as a function of the radial distance, yielding an individual rheology curve for each volumetric injection rate. This method excludes the near-well effects mentioned above and will provide local rheology curves for each injection rate, spanning different velocity intervals of the complete rheology curve.

**Figure 4:**
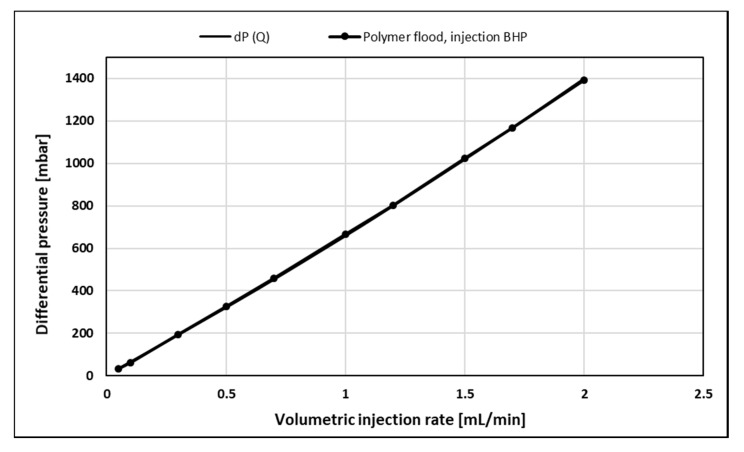
History match of injection BHP as a function of volumetric injection rate, dP(Q), for polymer flood.

**Figure 5:**
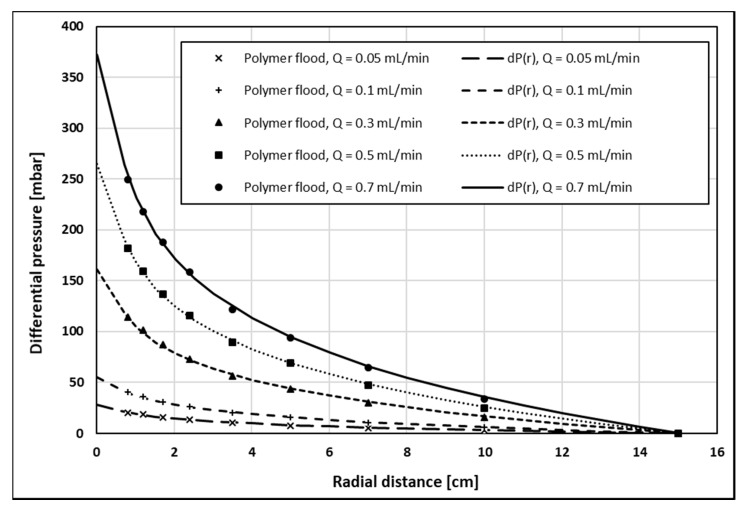
History match of internal pressures as a function of radial distance, dP(r), for volumetric injection rates of 0.05–0.7 mL/min.

**Figure 6:**
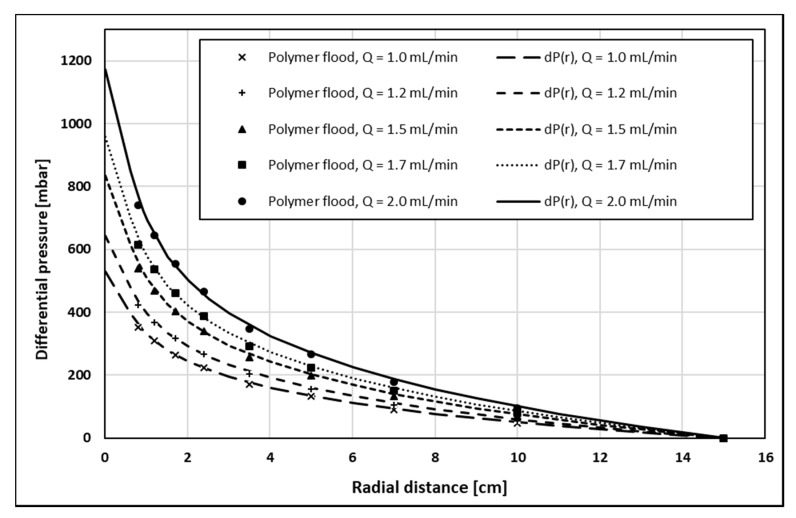
History match of internal pressures as function of radial distance, dP(r), for volumetric injection rates of 1.0–2.0 mL/min.

History match of injection BHP as a function of the volumetric injection rate (Figure 4) shows excellent agreement with the polymer pressure response. The history match error, defined in accordance with Gogarty, W.B. 1967, was 2.38% [14]. The history match of internal pressures as a function of radial distance (Figure 5 and Figure 6) also showed very good agreement with the polymer pressure response. Here, the average of history match errors was equal to 2.94%.

**Figure 7:**
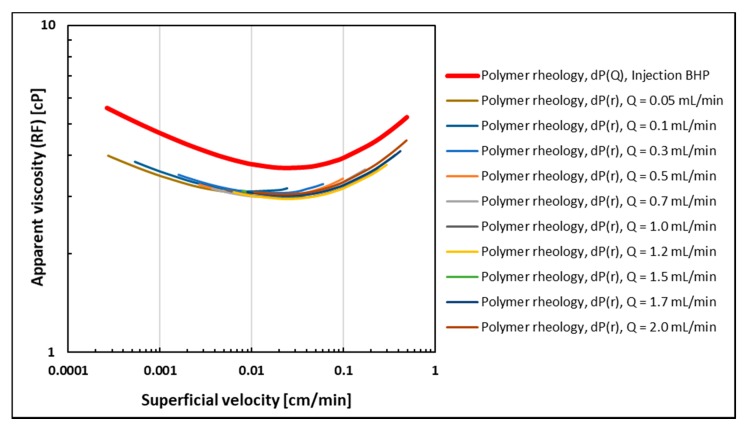
Polymer rheology curves obtained from history matching injection BHP as a function of the volumetric injection rate (red) and internal differential pressures as a function of radial distance (remainder of curves).

Polymer rheology curves obtained from both history match methods are shown in Figure 7. Since permeability obtained from the initial brine flood (before polymer flood) was used, apparent viscosity is represented by RF. Here, polymer rheology curves obtained by history matching differential pressure as a function of radial distance from the injection well are denoted dP(r). Using this history match method, individual rheology curves are obtained for each volumetric injection rate. However, since each curve was obtained at different injection rates, their maximum velocities at the injection point and their minimum velocity at the production rim were different. Rheology curves obtained at the lowest injection rates spanned the lower velocity interval, and were representing the shear-thinning rheology regime. In contrast, the highest injection rates spanned the higher velocity interval where the polymer behavior was increasingly shear-thickening. Even though they represent different injection rates and resulting velocity ranges of the polymer rheology, overlapping rheology curves were obtained, thus excluding the occurrence of rate effects. In addition, differential pressure was history matched as a function of the volumetric injection rate using injection BHP. This curve is denoted dP(Q) and shows the same shape as the remaining rheology curves.

Therefore, all polymer rheology curves show approximately the same functional relationship (shape) and two distinct flow regimes: Shear dominant flow is occurring at low to intermediate rates while extensional dominant flow is predominant in the high velocity regime. This rheology behavior is in accordance with the injection BHP build-up response where stabilization time was decreasing with injection rate, thus representing the transition from shear thinning behavior at a low rate to shear thickening behavior at higher injection rates.

The parallel shift between rheology curves obtained from injection BHP versus curves obtained from internal pressures is a consequence of performing history matches using the initial permeability (before the polymer is introduced). Since pressure measurements were conducted after retention was satisfied, permeability would be reduced both internally mainly due to adsorption, but also at a greater extent in the near wellbore region due to mechanical entrapment of polymer molecules. The greater local permeability reduction in the wellbore region versus the internal reduction would induce a higher pressure response for injection BHP and thus effectively shift the apparent viscosity to higher values due to retention effects. However, the consistency between the functional relationship obtained from injection BHP and internal pressures shows that injection BHP is a robust tool for estimating in-situ polymer rheology.

## 4. Field Scale Simulation Approach

A radial model was built using CMG STARS to simulate the field-scale polymer injection test at several injection rates for different in-situ rheology cases. The objective was to confirm the findings from the lab scale experimental and simulation studies in order to assess the design of polymer injectivity tests and define in-situ rheology signatures on BHP responses.

The model was used to test three different in-situ rheology behaviors: Shear thinning only, shear thickening only and shear thickening followed by shear thinning (combined). These three cases represent nearly all possible in-situ rheology behaviors expected in the near wellbore area of a polymer injector. The shear thinning case is representative for xanthan biopolymer, as well as types of synthetic polymers (such as HPAM) at certain low molecular weight and/or low concentrations, where shear flow dominates the in-situ behavior of polymer even at high shear [7]. In contrast, at certain high molecular weights or high concentrations, synthetic polymers might observe only shear thickening behavior in the near-wellbore region if they were dominated by extensional flow for the whole spectrum of encountered shear rates, shadowing the thinning behavior even at low shear rates. The third case (combined) represents the in-situ behavior observed in the lab for synthetic polymers where apparent shear thickening occurs at high shear rates followed by shear thinning away from the injection point.

Generic in-situ rheology curves were constructed using a modified version of the extended Carreau model introduced by Delshad et al. 2008 that relates apparent viscosity to Darcy velocity and includes both shear and extensional components [15]:

$$\mu_{app}=\left[\mu_{\infty }+\left[\left(\mu_{o}-\mu_{\infty }\right)*{\left[1+{\left(\lambda_{1}u\right)}^{2}\right]}^{\frac{n_{1}-1}{2}}\right]\right]+\left[\mu_{max}*\left(1-e^{-{\left[\lambda_{2}u\right]}^{n_{2}-1}}\right)\right]$$

where *μ_app_* is polymer apparent viscosity, *μ*_∞_ and *μ*_0_ are limiting Newtonian viscosities at high and low shear limits, respectively, *λ* and n are empirical polymer constants, *u* is the superficial velocity of the polymer in porous media and *μ_max_* is the shear-thickening plateau viscosity.

**Figure 8:**
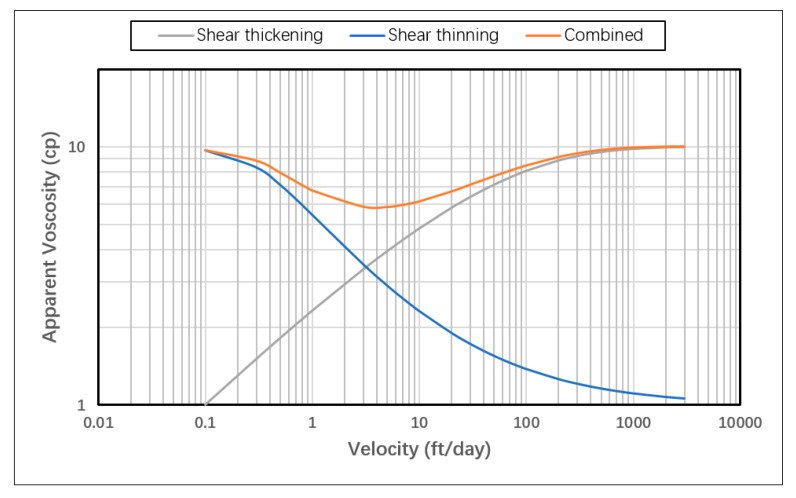
In-situ rheology curves obtained using extended Carreau model

**Figure A1:**
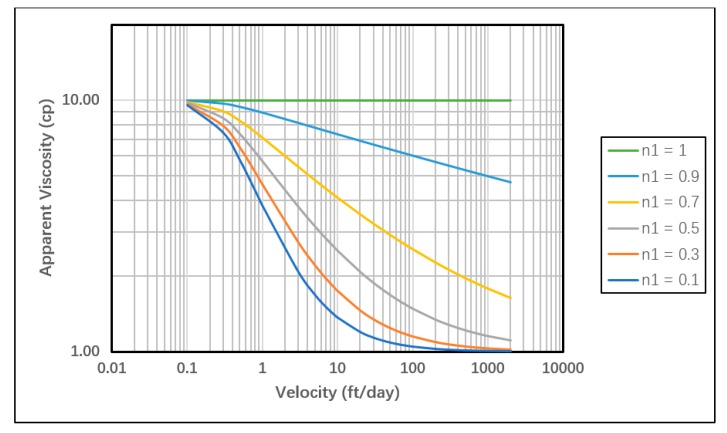
Sensitivity of shear thinning curve to different n1 values.

**Figure A2:**
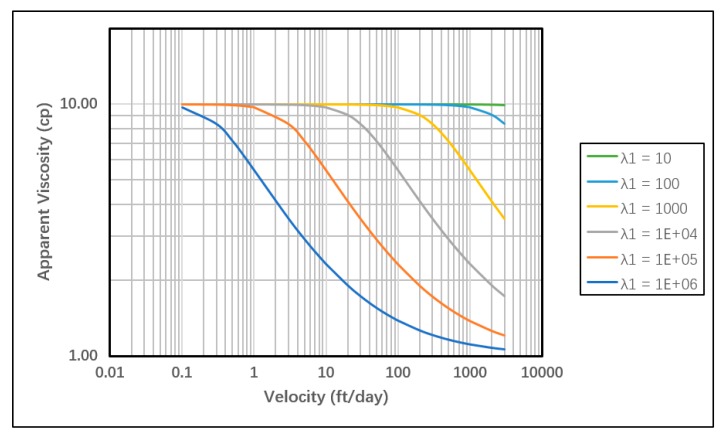
Sensitivity of shear thinning curve to different λ1 values.

**Figure A3:**
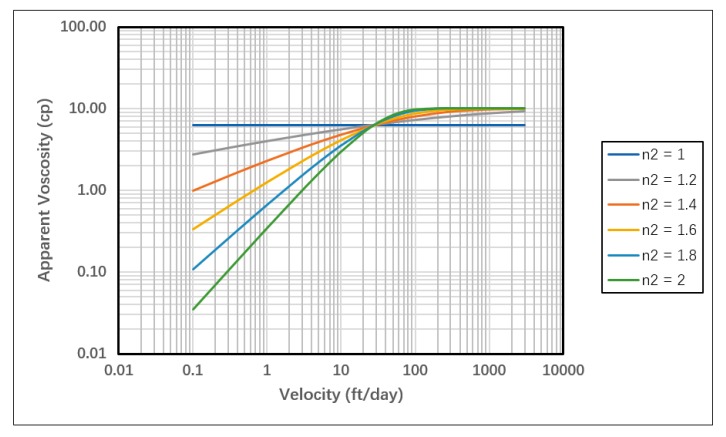
Sensitivity of shear thickening curve to different n2 values.

**Figure A4:**
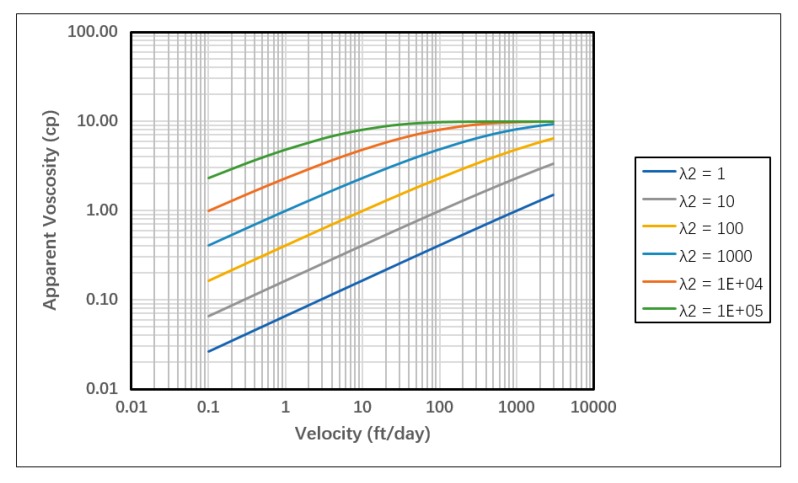
Sensitivity of shear thickening curve to different λ2 values.

Using this equation, apparent viscosity was calculated for the range of expected velocities in the near-wellbore region for all injection rates (Figure 8). The first part of the equation (shear flow component) was used for the shear thinning only case while the second part (extensional flow component) was used for the shear thickening only case. The sum of both parts was used for the combined rheology case. In order to ensure coverage of the entire expected velocity spectrum encountered in the reservoir, the model was first tested with the highest and lowest injection rates, then the velocity profiles were used as references for the rheology calculation. “$\mu_{\infty }$” was set at 1 cp since it represents pure solvent viscosity (water in our case). Other viscosity terms in the equation (*μ_max_* and *μ*_0_) represent the endpoints of the rheology curve and can be obtained from lab measurements with reasonable accuracy. Both were assumed at 10 cp in our study. *λ* and *n* parameters were tuned so that the curves are smooth and consistent for all rheology cases without compromising model stability. The sensitivity of shear thinning and shear thickening curves to *λ* and n parameters is illustrated in Figure A1, Figure A2, Figure A3 and Figure A4. Table 1 below shows a summary of the extended Carreau model parameters used for rheology curves.

### 4.1. Model Description

**Figure 9:**
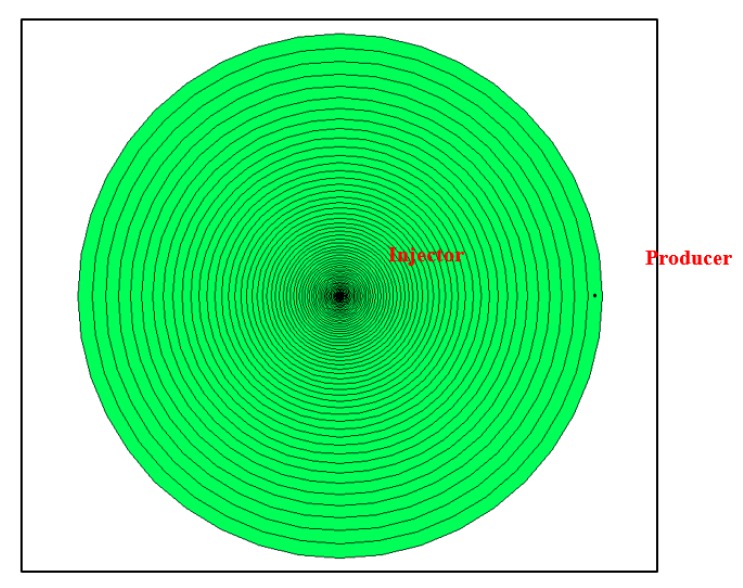
2D aerial map showing the radial gridding scheme of the model.

The radial coordinates system was selected to minimize the error in the velocity calculation induced by the smear of the velocity front in the Cartesian gridding scheme. A grid system of exponentially increasing grid size was applied for the near-wellbore area around the injector up to a 100 ft. radius (Figure 9). This gridding scheme was selected in order to accurately capture the expected exponentially decreasing velocity profile, and to improve simulation efficiency by avoiding unnecessary fine gridding further away from wellbore. The size of the innermost grid (injector grid) was set at 0.41 ft. while the size of the outermost grid was 5.58 ft. with a total of 60 grids in the radial direction.

This gridding system was generated automatically through the CMG Builder tool by defining a specific outer radius, the number of grids along the radius, and the size of the inner-most grid size (well grid). The arbitrarily selected grid sizing was based on the criterion of achieving sufficiently fine grids around the well-bore while maintaining model stability over all encountered viscosities at all injection rates. This is based on the fact that the finest-grid case represents the closest approximation to the realistic Darcy velocity at the wellbore sand-face and near-wellbore region, which is the only parameter that influences the predefined non-Newtonian viscosity functions that are inputted in the model as viscosity–velocity tables. Hence, grid sensitivity was not an issue of concern in this study.

Likewise, the model radius selection was subjective since it may not represent actual near-well bore area in many reservoirs as it varies widely based on reservoir properties. However, the radius was assumed at 100-ft to cover a wider spectrum of Darcy velocities/shear rates since the main objective is to inspect theoretical in-situ rheology impact on injection pressure rather than representing actual field cases. Therefore, the model was also assumed to be homogenous with no fractures or faults and saturated with water only.

Other factors that might impact BHP such as polymer adsorption and compressibility were not included as well. Polymer was injected at 800 ppm concentration represented by a 1.8 × 10^−6^ mole fraction input. Polymer adsorption was neglected and linear mixing rule was assumed between polymer and water viscosities. The range of tested rates is between 1000 and 10,000 bbl/day, which covers typical injection rates in field applications. The parameters of the model are summarized in Table 2 below.

### 4.2. Producers Pattern Sensitivity

**Figure 10:**
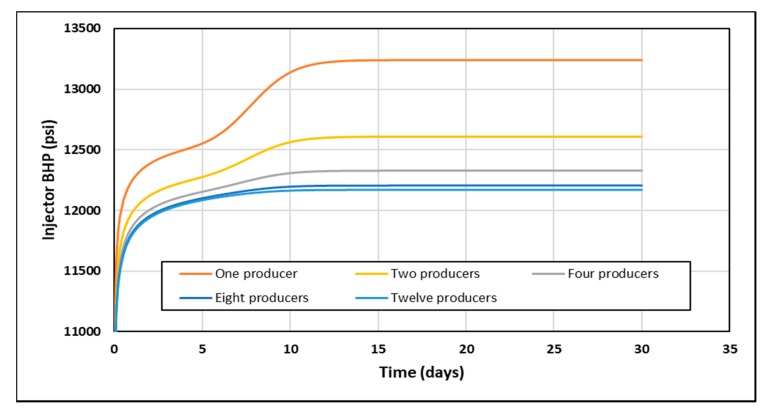
BHP response for each producer pattern at 6000 bbl/day with a shear thickening in-situ rheology.

**Figure A5:**
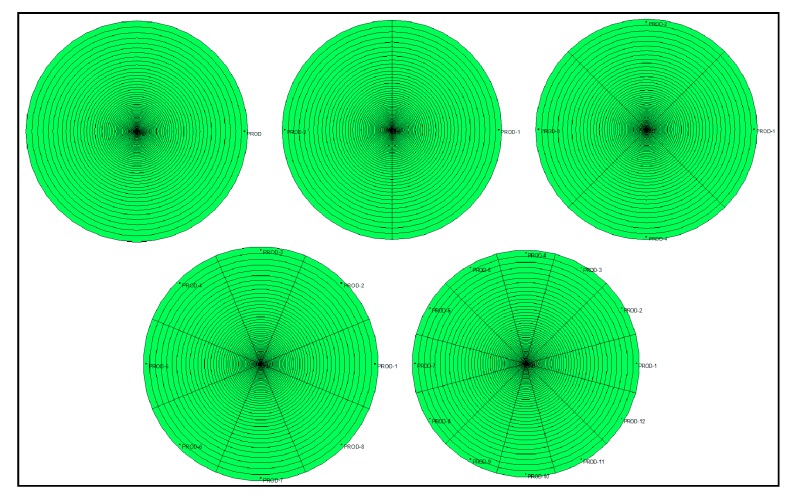
2D maps of producer pattern sensitivity cases.

A simple sensitivity study was performed to assess the impact of the number of producers on the simulation output in order to optimize the model’s pattern selection. The aim was to isolate the effect of polymer’s non-Newtonian viscosity by increasing the number of producers placed at the model’s outermost grids and thus eliminating a no-flow boundary impact on the injection pressure response. However, this may come at the price of increasing computation time by increasing the total number of grids in the model and hence lowering the simulation efficiency. The examined producer patterns were one producer only, two producers, four producers (five-spot), eight producers (nine-spot) and 12 producers. Figure A5 shows 2D maps of producer pattern sensitivity cases. Figure 10 shows the BHP response for each pattern under the conditions of an injecting polymer at 6000 bbl/day with a shear thickening in-situ rheology. The case with one producer shows a significant boundary effect after injecting 1 PV while the nine-spot and 12-producers patterns shows almost a no boundary effect. It takes 20 s to run the case with one producer compared to 120 s for the 12-producers case. It was decided that a nine-spot pattern was the most suitable for the purpose of this study, since the results were very close to the 12-produers case while simulation efficiency was not compromised significantly.

### 4.3. Viscosity Mixing in the Reservoir

**Figure 11:**
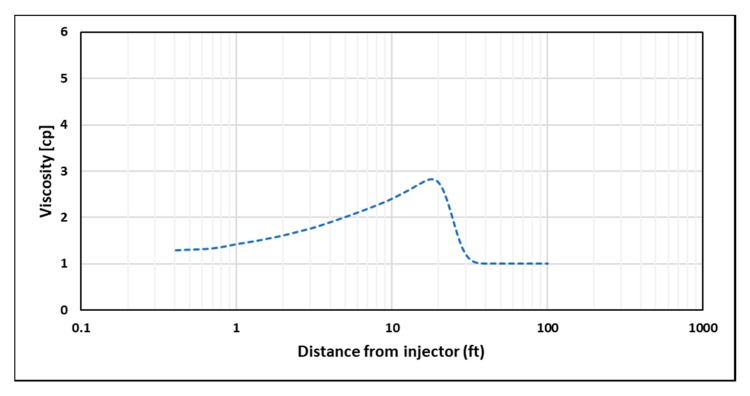
Viscosity profile after injecting 0.06 PV at 5000 bbl/day for shear thinning rheology.

**Figure 12:**
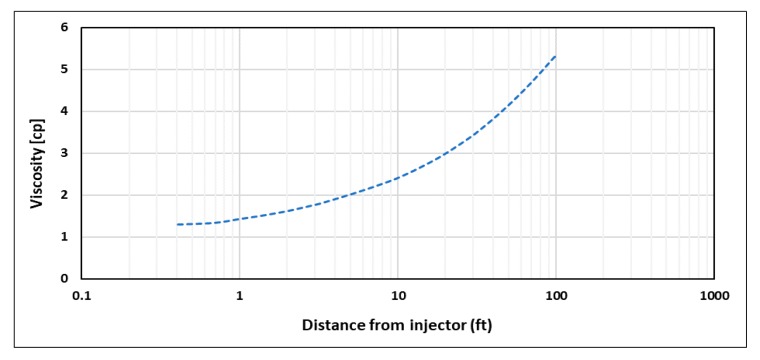
Viscosity profile after injecting 1 PV at 5000 bbl/day for shear thinning rheology.

One of the concerns when modeling polymer flooding is the mixing of polymer viscosity with other reservoir fluids viscosities, especially at the front of polymer slug. As the front is progressing, polymer viscosity behind it is following the predefined viscosity–velocity functions of the model. However, the viscosity ahead of the front will follow a viscosity mixing rule that creates a transition between polymer viscosity and reservoir fluid viscosity (Figure 11), and hence it would have an impact on the injector’s BHP that does not follow input viscosity functions. To isolate the non-Newtonian behavior effect on injector’s BHP, a minimum of 1 PV of polymer is required to be injected in order to achieve the intended viscosity profile within the near-wellbore region (Figure 12). In this study, STARS default linear mixing rule was applied between polymer and water viscosities.

### 4.4. Impact of High-Permeability Layers and Residual Resistance Factor

Heterogeneity of reservoirs adds more complexity to the challenges in determining in-situ rheology of a polymer from the injectivity test data. The presence of thief zones such as high permeability layers, open fractures, vuggy channels, etc., exposes the polymer to several different shear fields in the reservoir and creates the possibility of having several in-situ rheology behaviors occurring at the same time in different locations. Besides, the skin effect induced by polymer adsorption and/or mechanical entrapment in low permeable zones is another factor to be considered when tackling heterogeneity. The skin effect is usually addressed through the residual resistance factor (RRF), which is a parameter measured in lab core-floods and defined as the ratio between differential pressure after and before polymer injection.

**Figure 13:**
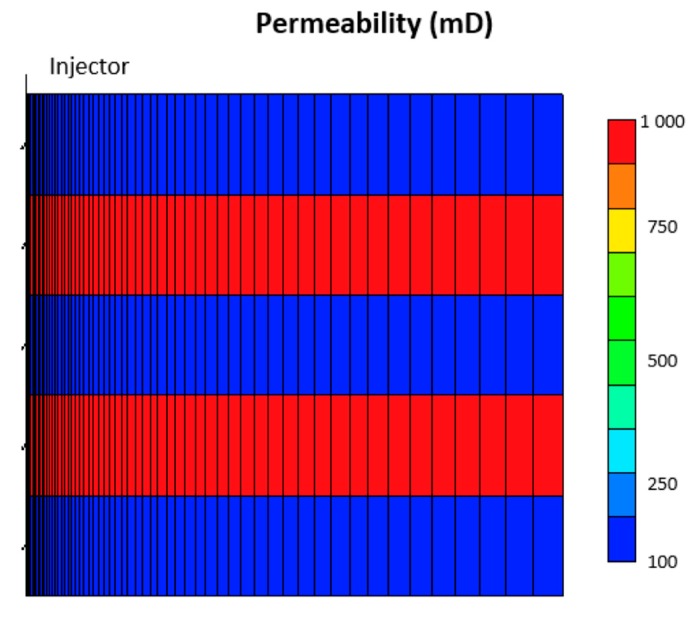
Cross section view showing the permeability distribution in the layered reservoir case.

To test the impact of different polymer in-situ rheology on layered reservoirs, the base model was modified to have alternating high and low permeability layers. High permeable zones were assigned a permeability value of 1000 mD, while low permeability zones were at 100 mD (Figure 13). The same rheology curves used for homogenous cases were used to ensure consistency of comparison. To test the impact of polymer adsorption and retention on low permeability zones, an extra case was investigated where an RRF value of two was set to the 100-mD layers while maintaining no adsorption (RRF = 1) in the high permeability layers.

## 5. Results and Discussion (Field Scale)

Results of simulations were used to plot stabilized BHP versus injection rate and time for homogeneous and layered cases at different in-situ rheology conditions.

### 5.1. Homogeneous Case

**Figure 14:**
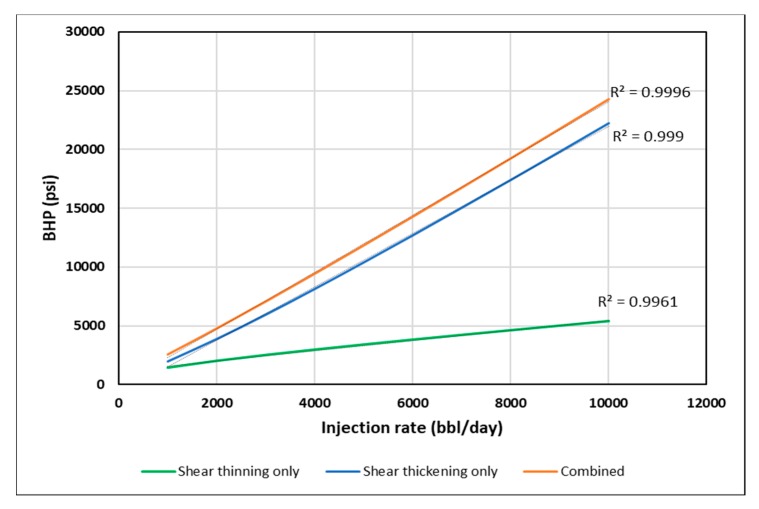
Stabilized BHP versus injection rate for different in-situ rheology in the homogeneous case.

Figure 14 shows BHP versus injection rate for the homogeneous cases. The BHP was fitted to a second order polynomial. The coefficient of second order term is positive or negative dependent on the type of rheology. It was found that stabilized BHP trend has an increasing slope for shear thickening only and a decreasing slope for the shear thinning only cases. The combined rheology showed a combination of increasing and decreasing slopes along the trend. This is attributed to the effect of shear thickening behavior for high shear rate near-wellbore, and shear thinning behavior for lower shear rates further away.

**Figure 15:**
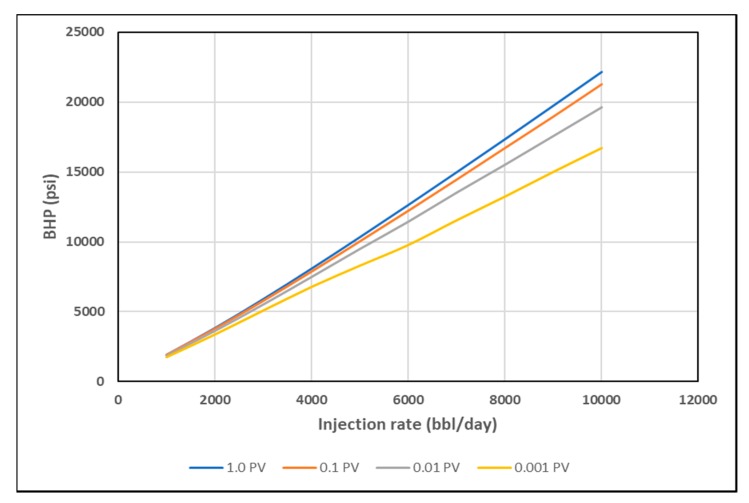
BHP vs. Q for shear thickening homogeneous case at different PVs.

**Figure 16:**
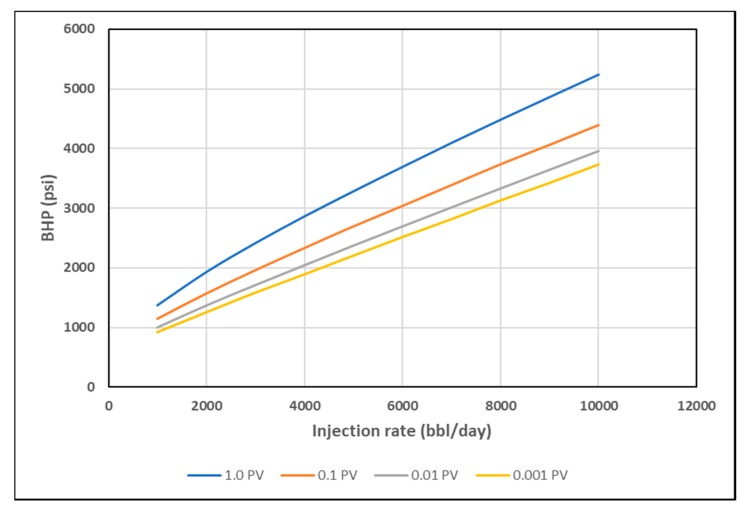
BHP vs. Q for shear thinning homogeneous case at different PVs.

**Figure 17:**
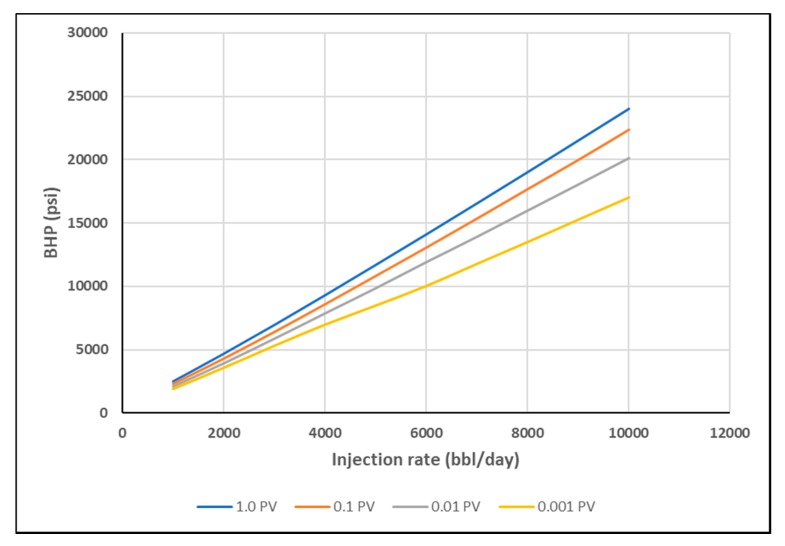
BHP vs. Q for combined rheology homogeneous case at different PVs.

As discussed earlier, stabilized pressure is not reached until at least 1 PV of the polymer is injected, as viscosity mixing at the front is eliminated and the steady-state condition is reached. This interval, however, is too long for polymer injectivity tests where rate steps are usually much shorter. Hence, BHP was plotted at 0.001 PV, 0.01 PV and 0.1 PV for each rate, to confirm if the same signal could be obtained at early times with transient condition and the presence of viscosity mixing. The slopes of BHP trends become less distinct with shorter injection times (Figure 15, Figure 16 and Figure 17). The slope change, however, is detectable by using the coefficient of second order polynomial trendline function (Table 3).

Negative values indicate shear thinning while positive values indicate shear thickening. For more detailed analysis of the coefficients, injection rates were divided into three ranges: low range from 1000 to 3000 bbl/day, medium range from 4000 to 7000 bbl/day and high range from 8000 to 10,000 bbl/day. From the obtained coefficients one can see that shear thinning and shear thickening behaviors can be detected by negative and positive values, respectively, even if shorter injection rate steps were implied. However, the behavior is more detectable as the injection time increases. The combined rheology is generally showing shear thickening behavior (positive values) due to the fact that apparent viscosity gain is the first encountered behavior in the near well bore area. The outliers that show positive for shear thinning and negative for shear thickening are attributed to the viscosity mixing phenomena.

Although the signal is not significantly pronounced in the BHP vs. Q plots, the slope change verifies the significance of rate-stepping in polymer injectivity tests. A minimum of three rate steps is thought to be sufficient in order to be able to detect in-situ rheology near-wellbore since it would yield two slope points that can indicate an increase or decrease in viscosity. The rates selected have to be selected so that they cover high, medium and low ranges of expected in-situ velocity.

**Figure 18:**
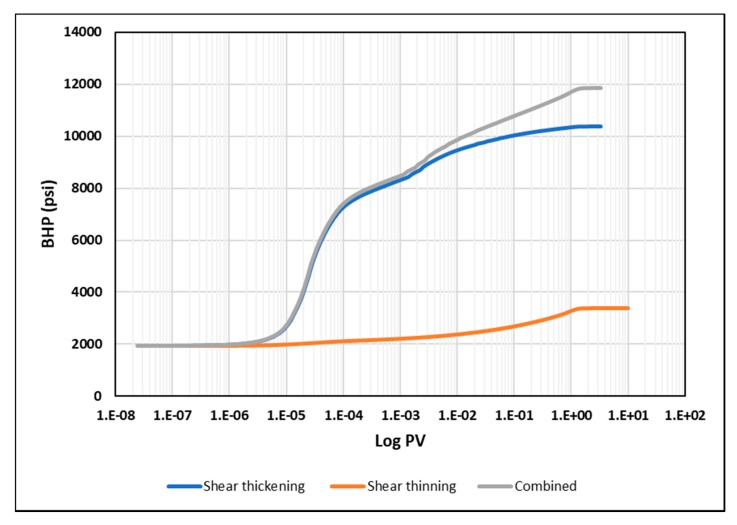
BHP versus log PV for different in-situ rheology at 5000 bbl/day in a homogeneous reservoir.

The second part of the analysis is focused on the BHP versus time plots. It was noticed that each in-situ rheology yields a distinctive signal during early times. Figure 18 shows, the BHP profile versus log time for each rheology at the injection rate of 5000 bbl/day. The shear thickening rheology reflects a sharp increase in BHP shortly after starting injection up to less than 0.005 PV. In contrast, shear thinning rheology is characterized by a gradual increase in BHP all the way to 1 PV. The combined rheology reflects a combination of the two behaviors of thickening and thinning. These signals are attributed to the viscosity that the injector “sees” first into the reservoir and then further away from it. These findings suggest that a minimum of 0.0001 PV of the near-well bore region may be sufficient to decide whether we encounter shear thickening or shear thinning rheology at the near wellbore area, however, the combined rheology would require longer periods of at least 1 PV to be detected through BHP versus time measurements. It is worth to note here that these findings are based on ideal case simulation results without considering other near well bore effects such as skin, fractures, filtrate cake, etc. Nevertheless, the suggested PV can be used as a base to analyze the BHP response near wellbore and to preassess in-situ rheology from early data obtained in the field.

### 5.2. Layered Case

**Figure 19:**
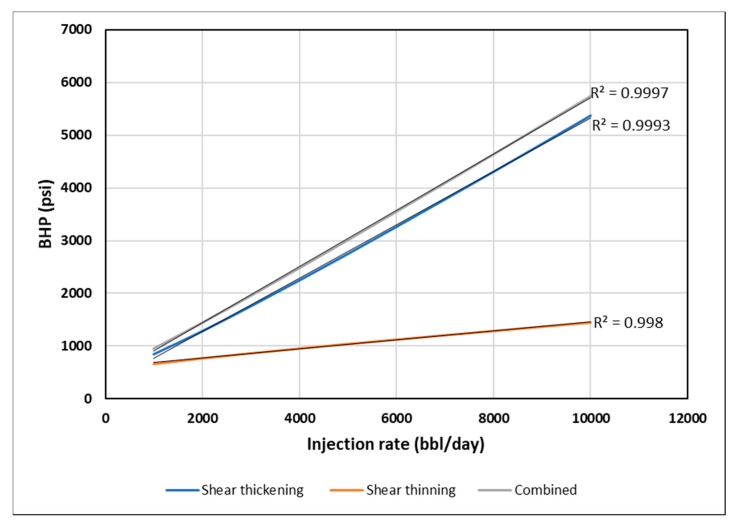
BHP versus injection rate for different in-situ rheology in the layered reservoir.

**Figure 20:**
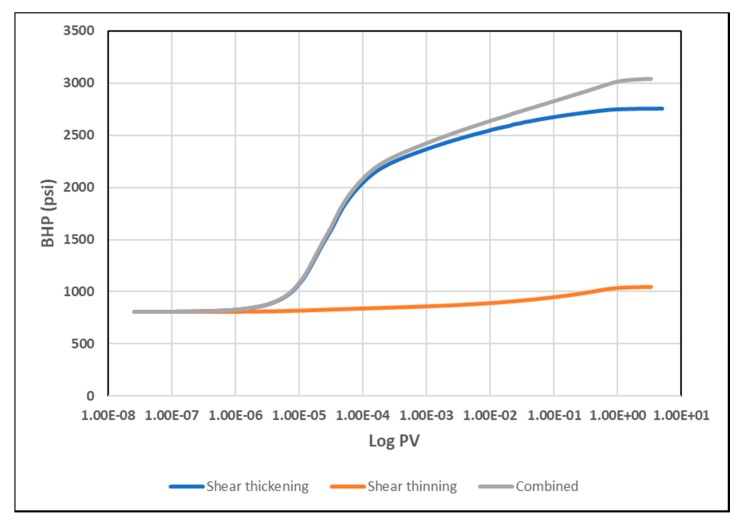
BHP versus log PV for different in-situ rheologies at 5000 bbl/day in the layered reservoir.

A basic heterogeneity case was investigated to observe the impact of high permeability streaks on the injection BHP compared to the homogeneous results. It is anticipated that high permeability layers can significantly enhance injectivity by lowering injection BHP. Nonetheless, we aim to find if heterogeneity can affect the distinctive signals of different in-situ rheology. The modified case is a layered reservoir with alternating high permeability (1000 mD) streaks (Figure 13). Figure 19 illustrates the BHP versus injection rate for the layered case. The trends of BHP responses are similar to the ones in homogeneous cases. This suggest that a similar method could be used for both homogenous and heterogeneous reservoirs. Besides, the signature on the BHP vs. log time is affected, and each rheology can be distinguished with the same characteristics observed in the homogenous case (Figure 20).

## 6. Conclusions

The findings presented in this study contribute to optimizing the design of polymer injectivity tests for enhanced oil recovery (EOR) polymer flooding projects. The main issue addressed was the impact of polymer in-situ rheology in porous media on the injector’s BHP response, since BHP is the main—and sometimes the only—source of data available in field tests. We suggest from this study that adapting a rate-stepped scheme in polymer injectivity tests is of a significant benefit in optimizing our understanding of polymer in-situ rheology at reservoir conditions and hence optimizing the design of polymer injectivity tests based on each individual reservoir characteristics.

The main conclusions are based on two simulation approaches: (1) history matching the results of a radial water and polymer flood lab experiments, (2) using generic up-scaled field-size model to test the impact of different in-situ rheology on BHP.

The experiment results showed that it is possible to distinguish between water and polymer floods based on BHP pressure build-up time. Moreover, the experiment confirmed the ability to differentiate between different rheological regimes based on BHP pressure-build up time, where shear-thickening behavior stabilizes pressure quicker than shear thinning. Lab-scale simulation findings confirmed the injection BHP as a robust tool for estimating in-situ polymer rheology in radial flow in porous media.

The field-scale simulation approach confirmed that BHP could be used to obtain information about in-situ rheology if rate variation is included in the procedure of field polymer injectivity tests. The rate-variation should include a minimum of three rate steps covering the whole range of velocities from low to high. Respective BHP readings then are used to determine the polymer rheology behavior as it propagates into the reservoir.

Besides the rate variation, it is important to assess the time the pressure needs to stabilize so it can be representative of the rheology signal. The findings suggest that the time for pressure stabilization at a given injection rate, is slower for shear-thinning fluids, compared to Newtonian and shear-thickening fluids. In addition, combined rheology exhibits a combination of shear thickening and shear-thinning behaviors that can be detected from BHP vs. time. Although pressure stabilization is affected by viscosity mixing at the front and hence it is not achievable before at least 1 PV is injected, the results confirmed that a minimum of 0.0001 PV could still be used to detect rheology signal. That being said, one can obtain information from a stepped-rate injectivity test only when comparing equal injected PVs for each rate. This finding is highly subjective to the specific assumptions of the model, but it can be used as a rough estimate to decide minimum injection duration at each rate step.

Furthermore, for heterogonous-layered reservoirs, it was found that the method is still applicable, despite the fact the rheological signal is noticeably reduced.

For future studies, we recommend investigating the impact of other near well bore effects such as skin, fractures, filtrate cake, etc. In addition, the impact of viscosity mixing needs to be quantified and further assessed in order to have better understanding of its impact on BHP response.

## Figures and Tables

**Table 1:** Extended Carreau model parameters used for rheology cases.

$\mu_{\infty }$	$\mu_{o}$	$\lambda_{1}$	$n_{1}$	$\lambda_{2}$	$n_{2}$	$\mu_{max}$
1	10	1.0 × 10^6^	0.46	1.0 × 10^4^	1.4	10

**Table 2:** Basic parameters and assumptions of the field scale model.

Parameter	Value
Well type	Vertical
Thickness	50 ft
Injector grid size	0.41 ft
Porosity	15%
Permeability	100 mD
Initial water saturation	100%
Reservoir pressure	2000 psi

**Table 3:** Coefficients of 2nd order polynomial trendline functions of BHP vs. injection rate at different injected PV’s for different rheology cases.

	Coefficient of 2nd Order Polynomial Trendline Function (×10^−5^)											
	0.001 PV			0.01 PV			0.1 PV			1.0 PV		
Rheology	High	Med	Low	High	Med	Low	High	Med	Low	High	Med	Low
**Shear thinning**	0.8	−0.4	−0.2	−0.03	−0.3	−1.0	0.3	−0.4	−2.0	−0.2	−0.5	-4.0
**Shear thickening**	−3.0	6.0	5.0	−2.0	2.0	6.0	3.0	2.0	7.0	6.0	2.0	8.0
**Combined rheology**	−1.0	6.0	3.0	0.9	0.5	6.0	4.0	1.0	5.0	−0.05	1.0	4.0
